# A Cybersecurity Culture Survey Targeting Healthcare Critical Infrastructures

**DOI:** 10.3390/healthcare10020327

**Published:** 2022-02-09

**Authors:** Fotios Gioulekas, Evangelos Stamatiadis, Athanasios Tzikas, Konstantinos Gounaris, Anna Georgiadou, Ariadni Michalitsi-Psarrou, Georgios Doukas, Michael Kontoulis, Yannis Nikoloudakis, Sergiu Marin, Ricardo Cabecinha, Christos Ntanos

**Affiliations:** 1 5th Regional Health Authority of Thessaly & Sterea, Mezourlo, 411 10 Larissa, Greece; fogi@dypethessaly.gr (F.G.); vstam@dypethessaly.gr (E.S.); atzi@uhl.gr (A.T.); kgounaris@ghv.gr (K.G.); 2 Decision Support Systems Laboratory, National Technical University of Athens, 15 780 Zografou, Greece; amichal@epu.ntua.gr (A.M.-P.); gdoukas@epu.ntua.gr (G.D.); mkontoulis@epu.ntua.gr (M.K.); cntanos@epu.ntua.gr (C.N.); 3 Department of Electrical & Computer Engineering, Hellenic Mediterranean University, 710 04 Heraklion, Greece; nikoloudakis@pasiphae.eu; 4 Polaris Medical Clinica de Tratament si Recuperare, Str. Principală, 407062 Suceagu, Romania; sergiu.marin@polarismedical.ro; 5 Hospital do Espírito Santo de Évora, EPE, Largo Senhor da Pobreza, 7000-811 Évora, Portugal; rjcabecinha@hevora.min-saude.pt

**Keywords:** cybersecurity culture, awareness, security assessment, healthcare domain

## Abstract

Recent studies report that cybersecurity breaches noticed in hospitals are associated with low levels of personnel’s cybersecurity awareness. This work aims to assess the cybersecurity culture in healthcare institutions from middle- to low-income EU countries. The evaluation process was designed and performed via anonymous online surveys targeting individually ICT (internet and communication technology) departments and healthcare professionals. The study was conducted in 2019 for a health region in Greece, with a significant number of hospitals and health centers, a large hospital in Portugal, and a medical clinic in Romania, with 53.6% and 6.71% response rates for the ICT and healthcare professionals, respectively. Its findings indicate the necessity of establishing individual cybersecurity departments to monitor assets and attitudes while underlying the importance of continuous security awareness training programs. The analysis of our results assists in comprehending the countermeasures, which have been implemented in the healthcare institutions, and consequently enhancing cybersecurity defense, while reducing the risk surface.

## 1. Introduction

Cybersecurity has become one of the dominant information technologies (IT) domains in the health sector [1]. Over recent decades, various scientific attempts have been made towards identifying, classifying, and addressing vulnerabilities and weaknesses in healthcare institutions and hospitals [2,3,4,5]. However, this effort did not discourage nor limit the continuously evolving cybercrime in this domain. The European Union Agency for Cybersecurity (ENISA) stated that the healthcare sector accounted for 27% of the overall cyberattacks in Europe in 2018 [6].

The coronavirus outbreak, among its many side-effects, resulted in a significant cybercrime increase [7,8]. Critical infrastructures, as categorized based on the 2016/1148 NIS Directive [9], have major targets. Among them, EU hospitals are experiencing patient data loss [10,11], ransomware, and availability attacks. The following are two of the most troubling examples:

- The Brno University Hospital in the Czech Republic which, on 12 March 2020, was forced to shut down its entire IT network, impacting two of the hospital’s other branches, the Children’s Hospital and the Maternity Hospital [12].
- A fatality in a German hospital linked to a cyberattack [13].

Although security infrastructure is of critical importance for the defense against cyber-criminals’ tactics and techniques, an organization’s biggest threat to privacy and security has been acknowledged to be its own personnel [14]. ENISA’s report in 2018 [6] revealed that 50.6% of attacked hospitals identified insider threats as their most serious adversary. 

As anticipated, a significant scientific effort has been made towards assessing healthcare personnel readiness over recent years [15,16,17]. Recognizing the multidisciplinary approach dictated towards this challenge, researchers soon adopted a holistic approach, and the term “cybersecurity culture” soon emerged. 

Cybersecurity culture denotes the combination of attitudes, behaviors, knowledge, and awareness the organization’s personnel display about common cyber risks and threats to protect the information assets [18]. Its evaluation involves the conduction of focused campaigns, which often results in the initiation of education programs, ICT infrastructure auditing, and the reassessment of current security policies to cultivate hospital personnel’s culture and sense of responsibility when processing sensitive information in daily business operations, thus preventing attacks or leakages [19,20]. Several endeavors towards assessing healthcare personnel’s cybersecurity culture were based on surveys. Indicatively, the surveys in Poland [21] and Finland [22] reported that medical professionals lack sufficient cybersecurity training. The analysis in [23] confirmed human error as one of the most common reasons for security incidents in hospitals. Authors in [24,25] highlighted that lack of security culture, awareness, and employee negligence or maliciousness constitute significant factors for the adoption of security policies.

Regarding the ICT resources utilization in hospitals, an analysis in 2008 [26] recorded a variation from 0.082 to 0.210 of ICT professionals (full-time job) per hospital bed in USA hospitals (0.142 in average, or equivalently, 1 ICT employee to total staff ratio of 60.7). Eurostat’s general report in 2018 [27] documented an EU average value of 3.9% for the relative share of ICT specialists in total corporate employment. 

This study analyses, before the COVID-19 situation, the overall disposition towards cybersecurity in healthcare institutions, which exhibit a proportion of ICT specialists in total employment below the EU average and compares the findings with relevant analyses in hospitals from Northern America and Northern Europe. We selected three different healthcare organizations from three different countries, i.e., Greece, Portugal, and Romania. In contrast to the above methods, we aimed to capture the cybersecurity awareness level of the organizations by first focusing on the ICT employees and consequently assessing the impact of this recorded level on the rest of the healthcare professionals.

The organizations under evaluation were the following: (i) a health region in Greece that comprises a significant number of reference hospitals and health centers (hereafter Institution A); (ii) a reference hospital in a large Portuguese region (henceforth Institution B); (iii) a Romanian medical clinic for impatient rehabilitation (henceforward Institution C). According to Eurostat [27], the percentage of ICT personnel to the total corporate staff for Greece was 1.8%, for Portugal it was 2.4%, and for Romania it was 2.2%. To the best of our knowledge, this is the first time that such an assessment has been conducted.

## 2. Methods and Materials

**Figure 1:**
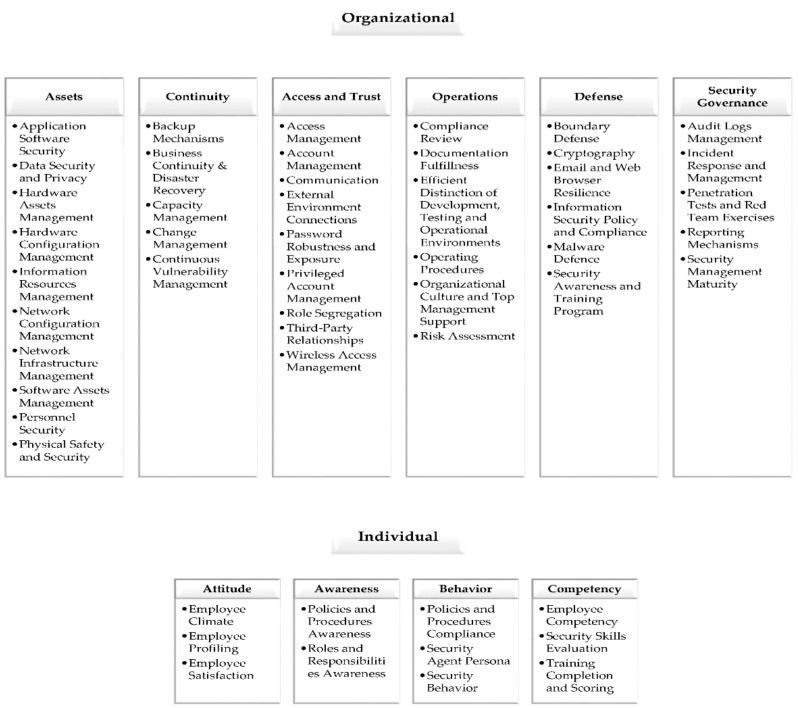
Cybersecurity Culture Framework.

The Cybersecurity Culture Framework was developed in 2019 in the context of the EnergyShield [28], a European Union (EU) project targeting cybersecurity in the electrical power and energy system (EPES). It was officially introduced to the scientific community in 2020 [29] in a manuscript detailing an evaluation methodology of both individuals’ and organizations’ security culture indicators. Its model consists of dimensions and domains analyzed into a combination of organizational and individual security factors (Figure 1). Thus, facilitating the assessment of organizational security policies and procedures in conjunction with employees’ characteristics, behaviors, attitudes, and skills. The specific framework exploits a variety of evaluation techniques, varying from surveys to more sophisticated approaches, such as simulations and serious games.

This study, using the aforementioned cybersecurity culture framework, aims to capture the perspective and the level of personnel’s cybersecurity awareness in the prior presented healthcare institutions. The percentage of the ICT staff compared to the total workforce is 0.45% for Institution A (average number among all the supervised units), 0.78% for Institution B, and 0.92% for Institution C (values lower than the Eurostat recorded statistics for these countries). The following two discrete online questionnaires were carefully designed to target two different personnel categories:

- Employees occupied in the ICT departments (ICT questionnaire);
- Non-ICT healthcare employees (non-ICT questionnaire) i.e., doctors, nurses, auxiliary, laboratory, and administrative personnel.

The survey’s questionnaires are presented in Appendix A and Appendix B, while the participation was on a voluntary and anonymous basis.

The ICT questionnaire comprised the following five parts: The first part included questions about demographics, years of experience, and serving population derived from the *Employee Profiling* domain of the *Attitude* dimension (individual level). The second part focused on ICT aspects involving the number of cybersecurity trainings performed, percentages of total budget allocation to ICT, and cybersecurity deriving from the *Security Awareness and Training Program* domain of the *Defense* dimension along with the *Security Management Maturity* domain of the *Security Governance* dimension (organization level). The Section 3 targeted computer network policies and external parties’ access combining indicators from different domains of the *Access and Trust* and *Assets* dimensions (organizational level). The fourth part requested individuals to answer questions about current cybersecurity methods and practices used deriving from the *Policies and Procedures Awareness* domain of the *Awareness* dimension (individual level). The last part focused on cybersecurity performance indicators (e.g., number of cyber security incidents over time and mean time for resolving an incident) deriving from the *Security Governance* dimension (organizational level). 

The non-ICT questionnaire included questions for demographics, employment status, cybersecurity, or related trainings such as General Data Protection Regulation (GDPR), the ability to understand cyberattacks, cybersecurity processes’ availability, and precautions taken. Security metrics were once again a combination of different indicators described in multiple layers of the cybersecurity culture framework aiming to obtain an overall evaluation of the non-ICT personnel culture. To sense if the non-ICT personnel had previously participated in cybersecurity campaigns, we used technical terminology in some questions of the questionnaire. In other words, several techniques were used to carefully trim and adjust the assessment process to the targeted audience.

The deployed numbers of computers are approximately 2800, 850, and 90 for Institutions A, B, and C, respectively. Knowing that it is generally difficult to voluntarily collect answers from the non-ICT personnel, due to the nature of their work, we sent the invitations (electronic and paper-based) to all the employees with the target to increase the response rate for the non-ICT personnel, and especially of those that have access to computers. Additionally, multiple-choice based questionnaires were translated from English to the native languages of the participants for better comprehension of their contents and to lift the language barrier and alleviate it from the equation. The collected data was translated back to English, harmonized, and checked for consistency. 

The surveys were conducted from September 2019 to November 2019. There was no time limit for the completion of the questionnaires and participants were not reimbursed or offered any other incentive. Furthermore, since our analysis focused on middle- to low-income countries, we conducted an extensive literature survey on evaluations of the cybersecurity awareness status of hospitals in the USA, Canada, and Northern Europe (high- to middle-income countries) so as to comparatively analyze our findings.

## 3. Results

**Figure 2:**
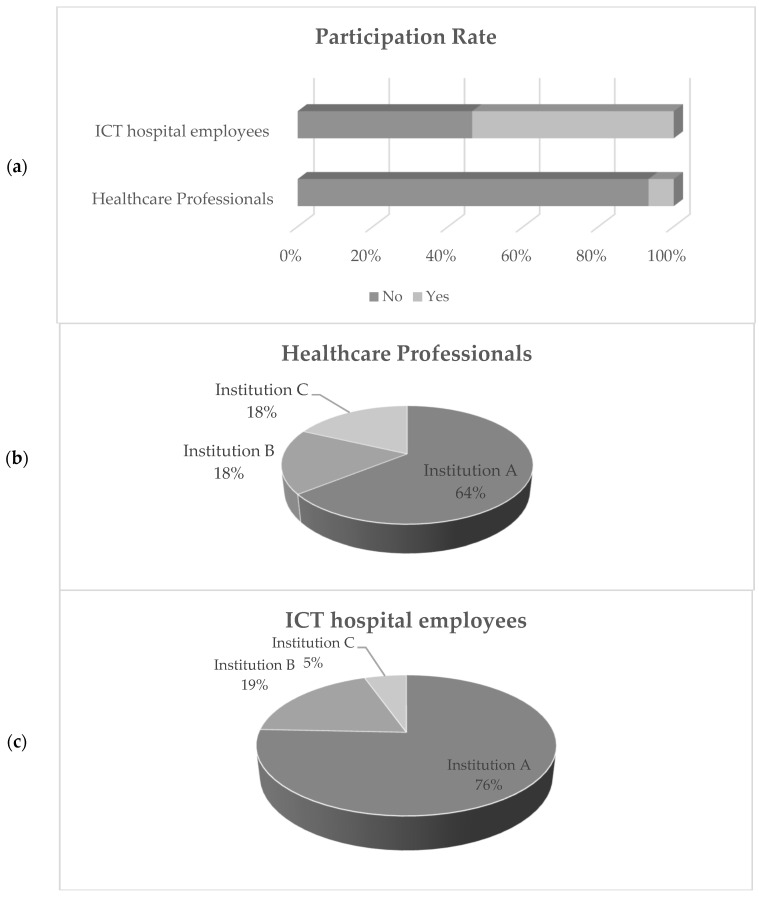
Campaign general participation information: (**a**) per profession, (**b**) healthcare professional per institution, and (**c**) ICT hospital employees per institution.

We invited 10,418 healthcare professionals (8500 from Greece, 1700 from Portugal, and 218 from Romania) and 69 ICT hospital employees (60 from Greece, 7 from Portugal, and 2 from Romania) to participate in the online survey. The participation rate is graphically presented in Figure 2. In total, 736 individuals responded to the surveys (37 for ICT and 699 for non-ICT). The overall answers to the ICT personnel were 28 (Institution A), 7 (Institution B), and 2 (Institution C), respectively, while for the non-ICT, the responses to the healthcare questionnaire were 449 (Institution A), 124 (Institution B), and 126 (Institution C). The response rate for the ICT personnel was 53.62%. The response rate for the non-ICT employees was 18.69% on the basis of the deployed computers and 6.71% on the basis of total employees, respectively.

### 3.1. Employed ICT Cybersecurity Procedures and Methods

The results revealed that 89%, 100%, and 50% of the ICT personnel in Institutions A, B and C, respectively, acknowledged the complete absence of dedicated cybersecurity departments in their institutions. Similar responses were given by the non-ICT personnel (86%, 63%, and 68% for Institutions A, B, and C, respectively). This deviation is due to the participants’ inability to distinguish between ICT and cybersecurity departments. In total, 100% of the ICT personnel in Institutions A and B and 50% in Institution C, responded they did not follow an incident response plan form responding to a data breach in a timely and cost-effective manner.

**Figure 3:**
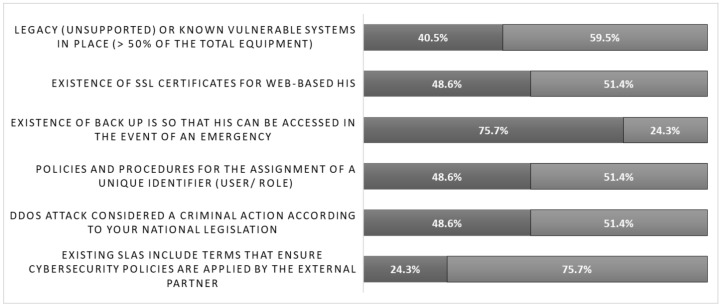
ICT personnel responses on common cybersecurity vulnerabilities.

The ICT questionnaire responses on common vulnerabilities (Figure 3) revealed they did not adopt common policies, irrespectively of their education status, gender, or age. Although obsolete and black-boxed technologies, deployed in hospitals, play a significant role in data breaches, 40.5% of ICT personnel indicated the usage of legacy systems with known vulnerabilities in their day-to-day operations (representing more than 50% of the total equipment). Additionally, only 24.3% were aware of the existence of cybersecurity terms within the service level agreements (SLA) with vendors. The importance of setting up a unique identifier policy for users and roles for the mitigation of the impact of internal threats was acknowledged only by 48.6% of the ICT personnel. The need for secure sockets layer (SSL) certificates to be used by the web-based health information system (HIS) was identified by only 48.6%. Only 48.6% of the ICT personnel were aware that certain attacks, such as distributed denial of service (DDoS), are considered criminal actions. On the other hand, 75.7% acknowledged the usage of proactive backup measurements.

**Figure 4:**
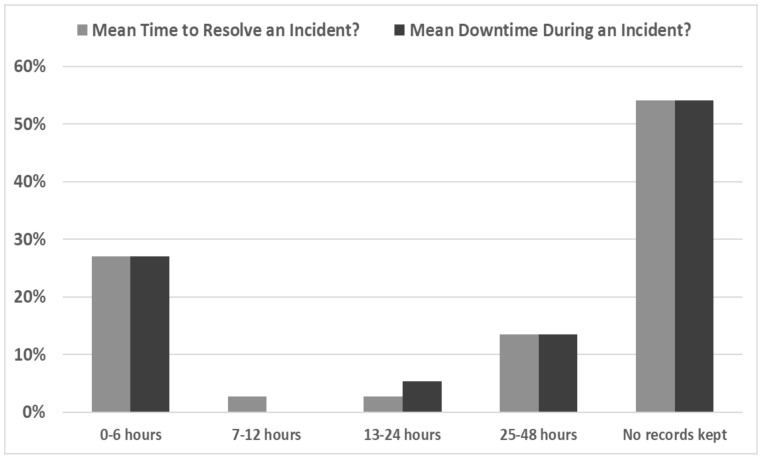
Cybersecurity Incident: Downtime and Time to Resolve.

Furthermore, 54% of the ICT personnel indicated that no records were kept, rendering a forensic analysis impossible and also resulting in no lessons learnt about the organizations’ response. Moreover, as shown in Figure 4, the ICT personnel replied that most identified cybersecurity incidents took up to 6 h to resolve. The analysis revealed that the “Mean Downtime” was equal to the “Mean Time to Resolve the Incident”, which means that parts of ICT facilities and related ICT-enabled services might have lost availability and functionality during the incident, a fact that possibly translates that no continuity plan was in place. This is in line with the finding that a small number of cybersecurity penetration tests were conducted during the last two years (affirmative answers: only 18% from A, 57% from B, and 50% from C). All the above indicate the necessity of performing regular ICT penetration tests and iterative trainings.

### 3.2. Training on Cybersecurity and Data Protection

**Figure 5:**
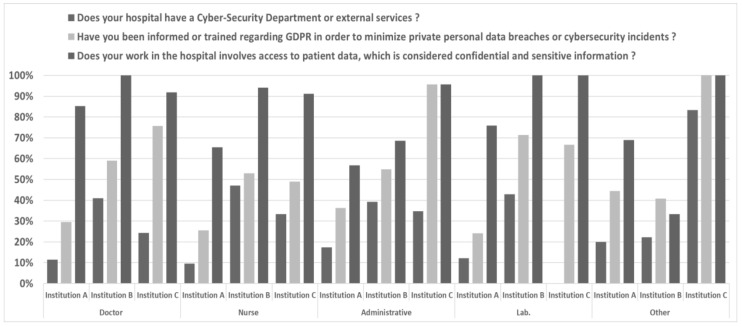
Awareness of Non-ICT personnel on legal aspects, privacy and cybersecurity structure.

The survey exposed the lack of cybersecurity-related training across the three institutions. 70% of the ICT personnel admitted they have not received official cybersecurity training in the past 3 years, with the remaining 30% revealing a frequency of less than one training per year even on European legislation and guidance, such as Directive (EU) 2016/1148 NIS Directive and the GDPR. Nevertheless, the ICT personnel responded they were aware of those acts at 80% in A, 86% in B and 50% in C. On the other hand, 73% of non-ICT personnel replied they had access to sensitive information and were aware of GDPR (Figure 5). 

39% of the ICT personnel in Institution A, 57% in Institution B and 100% in Institution C replied they used to perform internal cybersecurity awareness training (e.g., about phishing). The latter indicates the recorded low number of ICT staff might have played a significant role in not conducting training.

### 3.3. Cybersecurity Awareness Level

**Figure 6:**
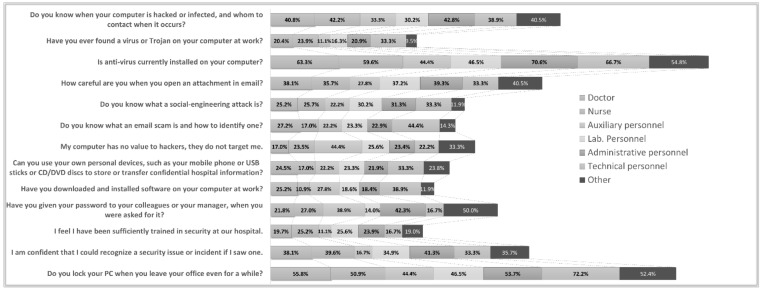
Digital behavior and security comprehension level of non-ICT Healthcare Employees.

Figure 6 shows the positive answers of the non-ICT group to questions related to cybersecurity awareness. Only 22.7% of the non-ICT personnel felt sufficiently trained in security, while only 38.5% were confident they could recognize a security issue or incident if they encountered one. This confidence was mainly supported by personnel in Institution C, while in the other two institutions they were perceived to be low-to-moderately trained. Trying to sensor the adequacy of the personnel’s awareness of cybersecurity threats such as email phishing and their reactions to them, it was found that only 26.8% of the participants knew what a social engineering attack was, while only 21.9% knew how to detect an email phishing attack. Although participants acknowledged they handled sensitive data on a daily basis, only 23.3% perceived the level of importance of their terminals’ content to hackers. 40.9% answered they knew when their terminals had been compromised and whom to contact in such a case. 30.9% understood the consequences of sharing their terminal or credentials, while 37.3% knew how to handle email attachments. More than 50% of the non-ICT personnel acknowledged the existence of antivirus software and the policy of locking their terminals when they leave. Their majority also responded (76%) that following security policies would help them do their job better.

## 4. Discussion

Our findings showed healthcare ICT personnel represented a very small percentage of the total workforce, generally below **1%**. The surveyed organizations have dedicated only a small amount of their total ICT budget (below **5%**) to cybersecurity purposes. The importance of budget allocation to cybersecurity is illustrated in the 2019 report [30] of the Healthcare Information and Management Systems Society (HIMSS) in the USA. Although our surveyed organizations reported less than **5%** in ICT budget allocation for cybersecurity, the report among **166** USA health information security specialists in [30] acknowledged a significant increase in this budget category (**10%** of the respondents acknowledged cybersecurity funding of more than **10%**, **11%** responded **7**–**10%,** while **25%** answered **3**–**6%**). The identified differences in the ICT investments indicate that smart hospitals have invested more in cybersecurity and in associated human resources to protect their information assets, rather than traditional hospitals [9], which are in the process of digital transformation. The ongoing application of the EU’s digital convergence policies (e.g., cross-border health data exchange) is expected to bridge the aforementioned gap.

Almost all of the respondents (**96%**) in the 2019 HIMSS survey [30] indicated their respective organizations conducted risk assessments (**37%** of which were comprehensive, resulting in the adoption of new or improved security measures by **72%** of them). In our study, the lack of cybersecurity departments and that **70%** of ICT employees have not received official cybersecurity training in the past **3** years, accounted for the low adoption and lack of standard or common policies in cybersecurity incidents (**100%** for A and B and **50%** for C). Only **48.6%** of the ICT personnel (A, B, and C) acknowledged the importance of applying a unique identifier policy for users and roles, in contrast to the 2017 survey [31] among **39%** of all the USA’s hospitals, where more than **90%** of them used unique identification for system users (supported by automatic logoff of system users, required use of strong passwords, etc.). Additionally, **40.5%** of the ICT personnel in our study indicated the usage of legacy systems (more than **50%** of the total equipment). The legacy-systems impediment is also acknowledged in [30] (**69%** of respondents), but in a lower percentage of the total equipment (more than **10%** for only **14%** of respondents). Moreover, in contrast to our study (see Section 3.2.), only **18%** of respondents in [30] stated their organization did not conduct phishing tests and trainings due to a lack of personnel with the appropriate cybersecurity knowledge and expertise. Furthermore, in our study, 21.9% acknowledged they do not know how to detect an email phishing attack, which suggests that the actual percentage of a real, ongoing phishing attack might be even higher. In contrast to that, a recent study at US health care institutions [15] indicates a median click rate on phishing campaigns of 16.7%, which is further reduced on subsequent ones, highlighting the importance of training on that matter. 

Regarding the non-ICT personnel, comparing our findings (Table 1) with a 2020 study in Poland [21], a 2019 study in a health region of western Finland [22], and a 2019 study in a health organization in western Canada [19], revealed the low level of cybersecurity awareness status. In Institutions A, B, and C, the **22.7%** that felt sufficiently trained in security and the **23.3%** that perceived the importance of the terminals’ content to hackers, appeared significantly lower next to the **51.31%** of the **1200** Finnish professionals reporting bring sufficiently aware of the information and the cybersecurity matters pertaining to their job. About the same percentage (**55.7%**) of **586** non-ICT professionals in the Canadian healthcare organization, declared their satisfaction with ICT security in their daily activities. **73.2%** of our non-ICT participants did not know what a social engineering attack was, which makes them a potential hazard of disclosing sensitive information (e.g., passwords). **69.1%** could not realize the consequences of sharing their terminal or credentials with other employees. The Finnish study reported a negative answer to disclosing one’s password over the phone, either if requested by an authority (**96%**), or by an ICT manager (83%), while the Canadian one states that **93.6%** would never share login information with other employees. Only **21.9%** of the respondents in our study could detect spam emails and phishing attacks, and only **37.3%** could handle attachments, which both fell behind the Finnish awareness score (**41.54%**) and the Canadian personnel that acted correctly upon them (**55%**). 

Low to moderate knowledge and awareness in the above fields pose a potential risk during daily working activities, such as processing patients’ data or communicating medical information to other parties. Therefore, it is deduced that there is a high risk of security incidents triggered by non-ICT employees because the aforementioned threats and attacks and the associated impact of potential incidents have not been efficiently communicated to them by the ICT staff. All the above indicate that decreasing the end-point complexity as proposed in [32], along with training conduction, is essential in raising awareness amongst personnel and motivating them to pay attention to cyber-threats and policies to limit human errors [33,34,35]. The adoption of a risk-aware attitude and associated skills by the non-ICT staff through cybersecurity trainings and a robust organizational monitoring strategy could lead to a more GDPR-compliant status. Even in New Zealand, a country where robust cybersecurity practices have long been in their agendas, the majority of internet users, as revealed by a 2019 survey [36], still take low to no security measures, while they perceive monitoring practices, such as the use of monitoring software, as highly technical. The necessity for intense cybersecurity awareness trainings, even from a young age, is highlighted.

## 5. Considerations and Limitations

The response rate of the non-ICT personnel was correlated with the number of the deployed computers they use daily. However, due to the variations in the clinic shift patterns, it was hard to stringently identify the non-ICT personnel that used computers. Therefore, we tried to collect responses by sending the non-ICT participation invitation mainly through their direct management, assuming it would be communicated to the total number of employees. Due to the voluntary and anonymous nature of the questionnaires, the commitment of all doctors and nurses was not totally ensured. Nevertheless, the collected answers proved to conform to the findings from the ICT questionnaire, where we managed to achieve high response rates. The availability of similar studies in the literature for the health care sector, especially recent ones, is not abundant, and comparisons were performed mainly against data from surveys conducted in hospitals in the USA, Canada, and some territories of northern Europe.

## 6. Conclusions

The implementation and deployment of security awareness programs in healthcare institutions along with training procedures proves to be a necessity. Furthermore, 76% of the non-ICT personnel replied that following hospitals’ security policies would help them perform their job better. Consequently, the findings were communicated to the management of the institutions, and certain courses of proactive and reactive cybersecurity measures have been triggered and implemented during the COVID-19 crisis. Specifically, a certain budget was allocated to procure or upgrade cybersecurity systems and software (e.g., antivirus databases, UTM firewalls with IDS/IPS). A specialised workshop has been conducted with ENISA’s support for the ICT staff, which in several cases was reinforced accordingly. Additionally, in-house awareness campaigns for non-ICT employees about anti-phishing or anti-social engineering have periodically been conducted. Moreover, those who deal daily with sensitive data and processes have participated in GDPR related seminars. In the future, we aim to revisit the updated cybersecurity measures and strategies and re-perform an extensive assessment to re-evaluate the new level of cybersecurity awareness and personnel readiness.

## Figures and Tables

**Table 1:** Percentage (%) of answers related to cybersecurity awareness along with the corresponding standard deviations for non-ICT personnel.

Question	Institution A *n* = 449 (100%)	Institution B *n* = 124 (100%)	Institution C *n* = 126 (100%)
Do you have cyber-security policies at your hospital?			
Yes	11% ± 0.5	55% ± 4.9	60% ± 5.3
No	14% ± 0.7	2% ± 0.2	7% ± 0.6
Do not know	75% ± 3.5	43% ± 3.8	33% ± 2.9
Have you been informed or trained regarding General Data Protection Regulation (GDPR) in order to minimize private personal data breaches or cybersecurity incidents?			
Yes	31% ± 2.5	31% ± 0.2	31% ± 0.1
No	69% ± 0.08	69% ± 0.2	69% ± 0.1
How careful are you when you open an attachment in email?			
I always make sure it is from a person I know, and I am expecting the email	32% ± 6.7	48% ± 15.9	50% ± 18.4
As long as I know the person or company that sent me the attachment, I open it	59% ± 7.7	42% ± 15.4	45% ± 18.4
There is nothing wrong with opening attachments	9% ± 6.3	10% ± 12.3	5% ± 7.4
Have you given your password to your colleagues or your manager, when you were asked for it?			
Yes	33% ± 9.1	26% ± 14.2	30% ± 24.1
No	67% ± 9.1	74% ± 14.2	70% ± 24.1
Is anti-virus currently installed on your computer?			
Yes	60% ± 2.8	16% ± 1.4	79% ± 6.9
No	11% ± 0.5	65% ± 5.8	5% ± 0.4
Do not know	29% ± 1.3	19% ± 2.7	17% ± 1.5
I am confident that I could recognize a security issue or incident if I saw one.			
Strongly agree	4% ± 2.4	4% ± 4.6	14% ± 12.3
Agree	24% ± 8.1	39% ± 18.3	59% ± 15.3
Neither agree nor disagree	42% ± 10	34% ± 18	8% ± 7.8
Disagree	23% ± 8.3	20% ± 9.1	17% ± 10.3
Strongly disagree	7% ± 4.5	3% ± 3	2% ± 1.9

## Data Availability

The data presented in this study are available on request from the corresponding author.

## Appendix A

### ICT Personnel Questionnaire

1. General Characteristics
  - Demographics
    - Age:
      20–39 ☐
      40–60 ☐
      60+ ☐
    - Gender:
      Male ☐
      Female ☐
  - Education:
    Secondary Education   ☐
    Vocational training Institution    ☐
    Bachelor’s Degree       ☐
    MSc             ☐
    PhD             ☐
  - Position:
    ICT director ☐
    ICT manager ☐
    ICT staff ☐
  - Years of experience
    0–5 ☐
    6–10 ☐
    more than 10 ☐
  - Healthcare Organization:
    Hospital ☐
    Clinic ☐
    Health Authority ☐
    National ☐
    Regional ☐
    Local ☐
      Employees <100 ☐
      Employees 100–300 ☐
      Employees 301–600 ☐
      Employees 601–1000 ☐
      Employees >1000 ☐
      Population <100 k ☐
      Population 100 k–300 k ☐
      Population >300 k ☐
2. Specific ICT
  - Proportion of ICT employees in total employment (%)
    0–1% ☐
    1.1–2% ☐
    2.1–3% ☐
    3.1–4% ☐
    4.1–5% ☐
    5.1–6% ☐
    6.1–7% ☐
  - Existence of a Cybersecurity Department
    Yes ☐
    No ☐
  - Official Trainings had in ICT cybersecurity during the last 3 years (number)
    0 ☐
    1 ☐
    2 ☐
    3 ☐
    4 ☐
  - Do you perform internal cybersecurity awareness trainings (e.g., phishing) in order to teach employees what to check in the received emails?
    Yes ☐
    No ☐
  - Average yearly organization’s budget allocated to ICT during the last 3 years (in Euros)
    0–100 K ☐
    101–200 K ☐
    201–300 K ☐
    301–400 K ☐
    401–500 K ☐
    Do not know ☐
  - Percentage of current ICT budget allocated to cybersecurity (e.g., antivirus purchasing or license renewal, firewall purchase or firewall license renewals, etc.) during the last 3 years (%)
    0–5% ☐
    6–10% ☐
    11–15% ☐
    16–20% ☐
    21–25% ☐
    26–30% ☐
    Do not know ☐
3. Network Communication with External Partners and Collaborators
  - Usage of secure method or other methods for third party accesses
    VPN ☐
    TeamViewer ☐
    AnyDesk ☐
    Remote Desktop ☐
    Other secure method ☐
    Other unsecure method ☐
  - Communication ports opened and monitored during daily operations (constantly or on demand)
    Port TCP 22 (SSH) ☐
    Port TCP 23 (Telnet) ☐
    Port TCP 3389 (RDP) ☐
    Port TCP 20 (FTP data) ☐
    Port TCP 21 (FTP control) ☐
    other ☐
  - Do existing SLAs include terms that ensure cybersecurity policies are applied by the external partner for preventing data breaches when connected remotely to hospital’s information systems?
    Yes ☐
    No ☐
    Do not know ☐
4. Cybersecurity Methods & Practices
  - Does your organization have an official cybersecurity plan?
    Yes ☐
    No ☐
    Do not know ☐
    If the previous answer is yes, which of the following plans?
      Risk Assessment ☐
      Incident Respond Plan ☐
      Mitigation plan ☐
      Report plan ☐
  - Have any cybersecurity tests been performed in your organization during the last 2 years?
    Yes ☐
    No ☐
    If the previous answer is yes, which of the following tests?
      Scanning ☐
      Penetration ☐
      Weak password identification ☐
      Phishing ☐
      Virus/malware checking ☐
      Verification of latest updates/outdates ☐
      Other ☐
  - Are you familiar with the Directive (EU) 2016/1148 NIS Directive and GDPR regulation?
    Yes ☐
    No ☐
    Partially ☐
  - Is a DDoS attack considered a criminal action according to your national legislation?
    Yes ☐
    No ☐
    Do not know ☐
  - Does your working practice have policies and procedures for the assignment of a unique identifier for each authorized user according to its role?
    Yes ☐
    No ☐
    Do not know ☐
  - Does your working practice have back up information systems so that it can access HIS in the event of an emergency or when your practice’s primary systems become unavailable i.e., in the event of a disaster?
    Yes ☐
    No ☐
  - Do SSL certificates exist for web-based hospital information systems?
    Yes ☐
    No ☐
    Partially ☐
  - Which of the following tools do you use daily for information security?
    Antivirus/malware ☐
    Firewall(s) ☐
    Data encryption (data in transit) ☐
    Data encryption (data at rest) ☐
    Patch & vulnerability management ☐
    Intrusion detection systems (IDS) ☐
    Network monitoring tools ☐
    Mobile device management ☐
    User access controls ☐
    Intrusion prevention system ☐
    Access control lists ☐
    Single sign on ☐
    Web security gateway ☐
    Multi-factor authentication ☐
    Data loss prevention (DLP application) ☐
    Messaging security gateway ☐
    Audit logs of each access to pt. health and financial records ☐
    My duties do not include cyber-security activities ☐
5. Cybersecurity Performance Indicators
  - Percentage of legacy (unsupported) or known vulnerable systems in place (e.g., end of life operating systems in medical devices) in total equipment (%)
    0–10% ☐
    11–20% ☐
    21–30% ☐
    31–40% ☐
    41–50% ☐
    51–60% ☐
    More than 60% ☐
    Do not know ☐
  - Number of cyber security incidents during the last 3 years (e.g., phishing attacks, virus infections, etc.)?
    0–5 ☐
    6–10 ☐
    11–15 ☐
    16–20 ☐
    21–25 ☐
    26–30 ☐
    No records kept ☐
  - Number of unauthorized login attempts in HIS, Active Directory, RIS/PACS per month?
    0–5 ☐
    6–10 ☐
    11–15 ☐
    16–20 ☐
    21–25 ☐
    26–30 ☐
    No records kept ☐
    No records kept but it is monitored regularly ☐
  - Mean time to resolve an incident?
    0–6 h ☐
    7–12 h ☐
    13–24 h ☐
    25–48 h ☐
    3–7 days ☐
    More than a week ☐
    No records kept ☐
  - Mean downtime during an incident?
    0–6 h ☐
    7–12 h ☐
    13–24 h ☐
    25–48 h ☐
    3–7 days ☐
    No records kept ☐

## Appendix B

### Non-ICT Personnel Questionnaire

1. General Characteristics
  - Demographics
    - Age:
      21–30 ☐
      31–40 ☐
      41–50 ☐
      51–60 ☐
      61+ ☐
    - Gender:
      Male ☐
      Female ☐
  - Education
    Secondary Education ☐
    Vocational training Institution ☐
    Bachelor’s Degree ☐
    MSc ☐
    PhD ☐
  - Position
    Doctor ☐
    Nurse ☐
    Auxiliary personnel ☐
    Lab. personnel ☐
    Administrative personnel ☐
    Technical personnel ☐
    Other ☐
2. Does your hospital have a cybersecurity department or external services?
  Yes ☐
  No ☐
  Do not know ☐
3. Does your work on the hospital involves working on a computer at any time?
  Yes ☐
  No ☐
4. Have you been informed or trained regarding General Data Protection Regulation (GDPR) in order to minimize private personal data breaches or cybersecurity incidents?
  Yes ☐
  No ☐
5. Does your work in the hospital involves access to patient data, which is considered confidential and sensitive information?
  Yes ☐
  No ☐
6. Do you have cybersecurity policies at your hospital?
  Yes ☐
  No ☐
  Do not know ☐
7. Do you know when your computer is hacked or infected, and whom to contact when it occurs?
  - Yes, I know when my computer is hacked or infected and I know whom to contact.
  - No, I do not know when my computer is hacked or infected and I do not know whom to contact.
  - Yes, I know when my computer is hacked or infected, but I do not know whom to contact.
  - No, I do not know when my computer and I know whom to contact.
8. Have you ever found a virus or trojan on your computer at work?
  - Yes, my computer has been infected before
  - No, my computer has never been infected
  - I do not know what a virus or trojan is
9. Is an anti-virus currently installed on your computer?
  - Yes
  - No
  - Do not know
10. How careful are you when you open an attachment in email?
  - I always make sure it is from a person I know, and I am expecting the email
  - As long as I know the person or company that sent me the attachment, I open it
  - There is nothing wrong with opening attachments
11. Do you know what a social engineering attack is?
  - Yes, I do
  - No, I do not
12. Do you know what an email scam is and how to identify one?
  - Yes, I know what an email scam is and how to identify one
  - I know what an email scam is, but I do not know how to identify one
  - No, I do not know what an email scam is or how to identify one
13. My computer has no value to hackers; they do not target me.
  - True
  - False
14. Can you use your own personal devices, such as your mobile phone or USB sticks or CD/DVD discs to store or transfer confidential hospital information?
  - Yes
  - No
  - Do not know
15. Have you downloaded and installed software on your computer at work?
  - Yes
  - No
16. Have you given your password to your colleagues or your manager when you were asked for it?
  - Yes
  - No
17. Which of these is closer to your thinking, even if neither is exactly right?
  - Following security policies at our hospital prevents me from doing my job
  - Following security policies at our hospital helps me do my job better
18. I feel I have been sufficiently trained in security at our hospital.
  - Strongly agree
  - Agree
  - Neither agree nor disagree
  - Disagree
  - Strongly disagree
19. I am confident that I could recognize a security issue or incident if I saw one.
  - Strongly agree
  - Agree
  - Neither agree nor disagree
  - Disagree
  - Strongly disagree
20. Do you lock your PC when you leave your office even for a while?
  Yes ☐
  No ☐
